# Awareness of Primary Biliary Cholangitis Among Turkish Physicians: A Cross-Sectional, Multicenter, Web-Based Survey

**DOI:** 10.3390/jcm15020915

**Published:** 2026-01-22

**Authors:** Hasan Eruzun, Henning Gronbaek

**Affiliations:** 1 Department of Gastroenterology, Samsun Training and Research Hospital, 55137 Samsun, Türkiye; 2 Department of Hepatology & Gastroenterology, Aarhus University Hospital, 8200 Aarhus, Denmark; henning.gronbaek@aarhus.rm.dk; 3 Department of Clinical Medicine, Aarhus University, 8200 Aarhus, Denmark

**Keywords:** primary biliary cholangitis, awareness, survey, Turkish, physicians

## Abstract

**Background:** Primary Biliary Cholangitis (PBC) requires early diagnosis and specialized management to prevent progression to cirrhosis. This study evaluates the awareness levels of Turkish physicians from various specialties regarding the clinical features, diagnostic criteria, and current treatment protocols of PBC. **Methods:** A multi-regional cross-sectional survey was conducted with 269 physicians across Türkiye. Knowledge levels were assessed through a 28-item instrument covering epidemiology, diagnosis and therapy. Data distribution was non-normal (Skewness: −1.296, Kurtosis: 2.857), necessitating the use of the Kruskal–Wallis H test and Dunn–Bonferroni post hoc procedure for inter-group comparisons. Internal consistency was confirmed with a Cronbach’s alpha of 0.80. **Results:** The overall mean awareness score was 62.6%. Item-level analysis revealed a near-universal understanding of the non-mandatory role of liver biopsy in diagnosis (99.1%) yet identified a critical knowledge gap regarding second-line therapies, particularly the use of steroids (6.8%). Significant disparities were observed among specialties (*p* < 0.001). Gastroenterologists (Median: 91.07%) and gastroenterology fellows (Median: 85.71%) exhibited significantly higher proficiency compared to general practitioners (64.29%) and family medicine residents (67.86%). Internal medicine specialists outperformed primary care providers, while no significant differences were found among other subgroups after Bonferroni adjustment. **Conclusions:** Professional specialization is the primary determinant of PBC awareness. While core diagnostic knowledge is stable, significant gaps exist in pharmacological management among non-specialists. Targeted medical education for primary care physicians is essential to ensure timely referral and optimize patient outcomes.

## 1. Introduction

Primary biliary cholangitis (PBC) is a chronic, non-suppurative liver disease of unclear etiology, primarily involving the small intrahepatic bile ducts. Although it predominantly affects women in their 5th–6th decades of life, it can occur in all age groups and in men. The main pathophysiological mechanism involves T cell-mediated destruction of intralobular bile ducts, leading to chronic cholestasis and, ultimately, chronic liver disease [1]. Pruritus and fatigue are the most common symptoms, significantly impairing patients’ quality of life [2,3]. While the incidence of PBC is highest in North America and Europe, wits prevalence is increasing worldwide, particularly in the Asia-Pacific region [4,5,6]. Although it is infrequently observed alongside other autoimmune liver diseases, the chronic nature of PBC, the existence of patient groups unresponsive to current treatments, and delays in diagnosis and treatment contribute to its clinical burden [7,8].

PBC often presents with elevated cholestatic enzymes such as alkaline phosphatase, which are common to many other clinical conditions. Therefore, detailed evaluation and clinical suspicion are crucial for diagnosis, necessitating a multidisciplinary approach in many cases [9]. Physicians across various specialties, from primary care providers to gastroenterology and hepatology specialists in tertiary centers, may encounter patients with PBC. Moreover, clinicians from different fields frequently interpret elevated alkaline phosphatase levels and order PBC-associated autoantibodies such as anti-mitochondrial antibody (AMA), AMA-M2, and anti-nuclear antibody (ANA) subgroups (e.g., SP100, GP210) [10]. Despite the availability of guidelines, meetings, and educational resources, awareness of PBC management may remain suboptimal even among gastroenterologists [11,12]. Also, for Türkiye, the low rate of AMA-negative PBC patients could be a sign of the low awareness of PBC [13].

In chronic diseases like PBC, requiring long-term follow-up, the physician–patient relationship, physician awareness, and continuity of care are vital for disease progression and treatment response [14]. While there are comprehensive studies on physician awareness of steatotic liver diseases in the literature, data on awareness regarding the diagnosis, treatment, and follow-up of PBC are exceedingly scarce [11,12,14,15,16]. Therefore, we designed an online survey to assess the awareness of physicians from various specialties most likely to encounter patients with PBC in Türkiye regarding the epidemiology, diagnosis, treatment, and management of PBC.

## 2. Materials and Methods

### 2.1. Participants

**Figure 1:**
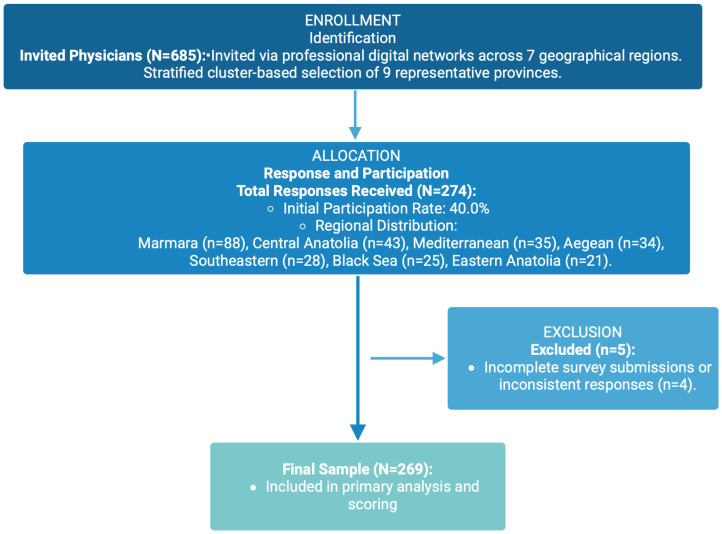
Flowchart of the study population. The recruitment process was designed to ensure proportional representation across the seven geographical regions of Türkiye.

A geographically stratified purposive sampling approach was employed to ensure a diverse and representative sample of physicians across Türkiye. To capture various clinical settings and socioeconomic backgrounds, nine representative provinces were selected from all seven geographical regions: İstanbul, Ankara, İzmir, Samsun, Rize, Kayseri, Mersin, Malatya, and Şanlıurfa (Figure 1). Within the specified regions, potential participants were identified via professional associations, unions, and relevant working groups, and the survey was distributed by email between 15 December 2024, and 15 February 2025. The survey’s informational text explicitly outlined the target audience and study objectives. Physicians who agreed to participate provided written informed consent and completed personalized surveys sent to their email addresses.

### 2.2. Data Collection

A total of 28 questions were developed, targeting physicians who were likely to encounter, diagnose, and manage patients with PBC. The questions covered the disease’s pathophysiology, epidemiology, diagnosis, treatment, and follow-up. Response options included “correct”, “incorrect”, and “do not know”. The questionnaire was developed based on two existing studies in the literature on PBC awareness [11,12]. The questions were restructured for physicians who are likely to play a role in the diagnosis and follow-up of patients with PBC in Türkiye. Before finalization, the questionnaire was reviewed and refined based on informal feedback from ten experienced gastroenterologists to improve clarity, clinical relevance, and face validity. No formal external validation process was performed. (Appendix A). The Google Forms platform was used to administer the survey. Responses could only be submitted once and were not modifiable. Responses were automatically recorded in the study coordinator’s email account upon submission. Participation was entirely voluntary, with no secondary incentives provided. At the beginning of the survey, the target audience, estimated completion time, and assurance that no personal information would be collected were clearly stated.

The survey instrument was divided into two distinct sections: demographic characteristics and awareness items. The awareness score was constructed and analyzed as follows:

The final awareness score was calculated based on 28 knowledge-related items. A binary scoring system was applied:

Correct answers: Assigned 1 point.

Incorrect answers: Assigned 0 points.

To ensure a conservative estimate of awareness and prevent guessing bias, all “I don’t know” responses were treated as incorrect and assigned 0 points. The raw scores were summed and then transformed into a standardized scale of 0 to 100 using the following formula:

$$\mathrm{Awareness}\;\mathrm{Score}=(\frac{\mathrm{Total}\;\mathrm{Sum}\;\mathrm{of}\;\mathrm{Correct}\;\mathrm{Items}\;}{\mathrm{Total}\;\mathrm{Number}\;\mathrm{of}\;\mathrm{Knowledge}\;\mathrm{Items}\;})\times 100$$

All items were weighted equally, and no reverse-coded items were used in the final composite score. As noted in the participant flow (Figure 1), a complete case analysis (listwise deletion) approach was adopted. Only participants who completed all knowledge items were included in the final scoring to ensure the robustness of the composite index.

To assess the internal consistency of the unvalidated survey tool, Cronbach’s alpha was calculated. A coefficient of 0.8 was obtained, indicating acceptable internal reliability. Furthermore, the distributional characteristics of the composite score (mean, median, and skewness) were evaluated to ensure the appropriateness of the non-parametric statistical tests used.

The survey design adhered to the guidelines outlined in The Checklist for Reporting Results of Internet E-Surveys (CHERRIES) [17].

Ethical approval for the study was obtained from the Samsun University Non-Interventional Clinical Research Ethics Committee (Decision Number: 2024/19/2, approval date: 23 October 2024).

### 2.3. Statistical Analysis

Data are reported as percentage or median with interquartile range (IQR). Pearson chi-square test was used in intergroup comparisons of categorical variables. The distributional characteristics of the awareness scores were evaluated to determine the appropriate statistical tests. The skewness (−1.296) and kurtosis (2.857) values indicated a significant departure from normal distribution. Consequently, non-parametric tests (e.g., Kruskal–Wallis) were utilized for group comparisons as they are more robust to violations of normality. Post hoc analyses were performed with Dunn–Bonferroni test. Descriptive statistics were used for the percentages of the variables. A *p*-value of 0.05 represented statistical significance. Statistical analysis was performed in IBM SPSS Statistics for Macintosh, Version 26.0. IBM Corp; 2019. Armonk, NY, USA.

## 3. Results

The survey had a participation rate of 40%, and 90% of the participants reported being familiar with PBC. There were no major differences between responders and non-responders for age, gender, specialties and working place. Most participants were aged between 31 and 35 years and were physicians working in university hospitals (Table 1). Among specialties, internal medicine specialists and residents constituted the largest group, while family medicine specialists were the least represented. Detailed demographic data are provided in Table 1.

The performance of participants on individual survey items showed a wide range of variation (Table 2). The overall mean correct response rate across all 28 items was 62.6%.

The highest level of awareness was recorded for the non-mandatory nature of liver biopsy in diagnosis (Q18: 99.1%) and the fact that PBC occurs in both genders (Q3: 83.8%) and its multi-organ association (Q6: 83.8%).

The most significant knowledge gap was identified regarding the use of steroids in treatment (Q25: 6.8%). Additionally, a substantial number of participants struggled with the clinical specificities of male PBC prevalence (Q4: 28.2%) and the systemic nature of the disease (Q7: 28.2%).

**Figure 2:**
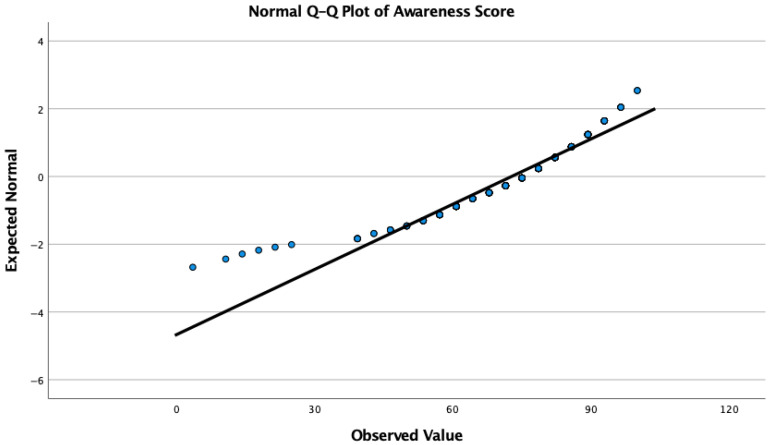
Distribution of the total awareness score.

The median awareness scores were compared across different medical specialties and titles to identify professional disparities in knowledge levels. Given the non-normal distribution of the total awareness scores (Skewness: −1.296, Kurtosis: 2.857), a Kruskal–Wallis H test was employed for group comparisons (Figure 2). The analysis revealed a statistically significant difference in awareness levels among the professional groups (χ^2^ (9) = 63.81, *p* < 0.001).

The awareness scores were further analyzed across different medical specialties to identify subgroup-specific variations. As summarized in Table 3, the central tendency and dispersion of scores varied significantly among professional categories.

Gastroenterologists demonstrated the highest level of proficiency with a median score of 91.07% (Mean ± SD: 89.28 ± 10.32), followed closely by gastroenterology fellows with a median of 85.71% (84.34 ± 6.60). In contrast, the lowest median scores were observed among family medicine specialists (60.71%) and general practitioners (64.29%), highlighting a notable disparity in specialized knowledge.

Internal medicine specialists and residents showed moderate awareness levels, with median scores of 78.57% and 75.00%, respectively. The relatively wide standard deviations and interquartile ranges (IQR) observed in groups such as general surgery residents (SD = 28.08) suggest a high degree of intra-group variability in awareness levels compared to the more consistent performance of the gastroenterology-focused cohorts.

To address the potential for Type I errors in multiple comparisons, a post hoc analysis was performed using the Dunn–Bonferroni procedure. As summarized in Table 4, gastroenterologists (Median: 91.07%) and gastroenterology fellows (Median: 85.71%) demonstrated significantly higher awareness scores compared to general practitioners (Median: 64.29%, Adjusted *p* < 0.001) and family medicine residents (Median: 67.86%, Adjusted *p* = 0.001). Additionally, internal medicine specialists (Median: 78.57%) exhibited significantly higher scores than general practitioners (Adjusted *p* = 0.021). No significant differences were observed between other professional groups after applying the Bonferroni adjustment (*p* > 0.05).

Correct response rates were significantly higher among physicians undergoing specialty training and those with more than ten years of professional experience (Table 5). No significant differences were observed in correct response rates based on gender or employment in university hospitals (Table 5).

## 4. Discussion

This study aimed to evaluate the awareness of PBC diagnosis, treatment, and follow-up among physicians from various specialties and hospitals, who are likely to encounter and manage patients with PBC. Our findings demonstrate that awareness of PBC was highest among gastroenterologists and gastroenterology fellows. Physicians who had completed specialty training and those with over ten years of professional experience also exhibited significantly higher awareness levels. However, awareness of specific aspects, such as the role of non-AMAs in diagnosis, evaluation of the response to UDCA therapy within the first year and the absence of steroids in PBC treatment, was notably low across most groups. Additionally, general awareness regarding PBC management and follow-up was found to be suboptimal. These results are similar to a Chinese study regarding the awareness of PBC [12].

The rarity and indolent course of PBC likely contribute to its low awareness among physicians, especially family physicians and general practitioners. However, a lack of awareness may exacerbate the disease burden due to delays in diagnosis, failure to detect treatment unresponsiveness early, and missed opportunities to recognize complications. This underscores the need for greater diagnostic consideration and appropriate testing in patients presenting with elevated liver enzymes, particularly by family physicians, internal medicine specialists, and general surgeons. In Türkiye, while the prevalence of chronic liver diseases secondary to autoimmune conditions has remained stable, the increasing population has led to a growing number of newly diagnosed patients with PBC each year [18].

Our findings also indicate that awareness significantly improves with specialty training across all fields. Türkiye has a well-established system for medical specialization, with competitive national exams determining admissions and training durations of 4–5 years, or longer for subspecialties such as gastroenterology [19]. Despite a physician-to-population ratio below the OECD average, the diverse case exposure during training may enhance clinical expertise [20,21]. Although there are no prior studies directly investigating the impact of specialty training on PBC awareness, a study from India showed that consultants and specialists in internal medicine demonstrated higher awareness of NAFLD diagnosis and management compared to residents [22]. Similarly, awareness of PBC was significantly higher among physicians aged over 35 and those with specialty training in our study, highlighting the positive impact of clinical experience and advanced training.

Notably, no significant difference was observed in awareness levels between physicians working at university hospitals and those in other centers. Although we could not analyze physicians in private practice due to the low number, previous studies from France have indicated that physicians in private clinics had lower awareness of chronic liver diseases compared to those in public or university hospitals [23]. Our findings suggest comparable awareness levels among physicians in public and university hospitals in Türkiye.

While the risk of hepatocellular carcinoma (HCC) in PBC patients is lower than in other chronic liver diseases, it is significantly higher than in the general population, particularly in patients at an older age, male patients, and in patients with cirrhosis [24]. Awareness that PBC can affect men was satisfactory across all specialties; however, awareness of the risk of HCC in PBC patients was lacking among more than half of family medicine specialists. This could hinder early detection of HCC in patients with PBC.

Non-AMAs, such as anti-gp210 and anti-sp100, play a role in PBC diagnosis and are associated with a worse prognosis [25,26]. Awareness of these antibodies was low across all specialties except gastroenterology, underscoring the need to improve knowledge of PBC-specific ANA subtypes. Evaluating the response to UDCA therapy within the first year using validated scoring systems is critical for determining disease prognosis [27]. While awareness of this was relatively good among internal medicine specialists, it was alarmingly low among family physicians (20%). Awareness of second-line treatments beyond UDCA, however, was satisfactory across all specialties.

Steroids are not used in PBC management outside overlap syndromes due to their inefficacy and side effects [28]. Surprisingly, awareness of this was below 50% across all specialties, including gastroenterologists, suggesting potential misconceptions stemming from PBC’s autoimmune association. While awareness of osteoporosis management was good among primary care physicians in Germany [29], our study showed that awareness of the need for bone mineral density testing in patients with PBC was low among family medicine specialists and general practitioners (40%). This highlights the need to increase osteoporosis-related awareness among primary care providers for patients with PBC in Türkiye. It could be beneficial to prepare some guidelines about chronic liver diseases such as PBC, for primary care physicians.

A major strength of our study is the inclusion of physicians familiar with PBC from across Türkiye and from different specialties. However, this study has several limitations that should be acknowledged when interpreting the results. First, the cross-sectional and web-based nature of the survey may have introduced a selection bias, as physicians who are more digitally active or have a specific interest in hepatology might have been more likely to participate. Second, while the participants represent nine different provinces, a significant portion of the sample consists of physicians working in tertiary care university hospitals. This overrepresentation of academic settings may not fully reflect the awareness levels in primary and secondary healthcare facilities across the country. Therefore, our findings should not be interpreted as a definitive ‘national awareness level’ but rather as an indicative assessment within the studied cohort. Additionally, the relatively young age profile of the participants and the inherent limitations of self-reported data might influence the generalizability of the results. Future research involving a more stratified and larger sample size, including a broader representation of rural and secondary care settings, would be beneficial to provide a more comprehensive overview of PBC awareness among all Turkish physicians.

## 5. Conclusions

Our study demonstrates the need to improve awareness of PBC diagnosis, management, and follow-up, particularly among primary care physicians in Türkiye. In addition, efforts should focus on increasing awareness of the timely evaluation of UDCA therapy response, a critical determinant of disease progression, among non-gastroenterologists managing PBC patients. Targeted educational programs, training initiatives and national guidelines could play a pivotal role in addressing these gaps and improving patient outcomes.

## Figures and Tables

**Table 1:** Demographic and Professional Characteristics of the Participants (n = 269).

Variable	n	%
Age (years)		
20–25	19	7.0
26–30	85	31.5
31–35	89	33
36–40	36	13.3
41–45	21	7.8
46–50	11	4
51–55	4	1.4
56–60	4	1.4
Gender		
Female	130	48.3
Male	139	51.6
Workplace		
Public hospital	78	28.9
University hospital	174	64.6
Private hospital	12	4.4
Family medicine office	5	1.8
Specialty		
General practitioner	25	9
Internal medicine trainee	64	23.7
Internist	89	33
General surgery trainee	10	3
General surgeon	10	3
Family medicine trainee	19	7.0
Family medicine specialist	6	2
Gastroenterology fellow	13	4.8
Gastroenterologist	18	6.6
Other fellows	15	5.5

Data are presented as numbers (percentage).

**Table 2:** The performance of participants on individual survey.

Question Number	Correct Response Number	Correct Response Rate (%)	Awareness Level *
Q1—PBC was formerly known as primary biliary cirrhosis.	88	75.2%	Good
Q2—PBC is more common in women.	81	69.2%	Good
Q3—PBC does not occur in men.	98	83.8%	Very good
Q4—The prevalence of PBC in men is increasing.	33	28.2%	Low
Q5—The prognosis in men is worse than in women.	63	53.8%	Medium
Q6—Other autoimmune diseases can be seen in PBC	98	83.8%	Very good
Q7—PBC is a liver-specific disease	33	28.2%	Low
Q8—PBC is associated with large bile ducts.	69	59.0%	Medium
Q9—PBC is associated with small intrahepatic bile ducts.	66	56.4%	Medium
Q10—PBC is an autoinflammatory liver disease.	78	66.7%	Good
Q11—PBC does not lead to cirrhosis.	88	75.2%	Good
Q12—PBC does not cause hepatocellular carcinoma.	75	64.1%	Good
Q13—PBC does not cause liver failure.	84	71.8%	Good
Q14—AMA positivity plays a critical role in PBC diagnosis.	90	76.9%	Good
Q15—There is no other antibody specific for PBC diagnosis.	60	51.3%	Medium
Q16—Fatigue and itching are the most common symptoms of PBC.	87	74.4%	Good
Q17—AMA positivity is mandatory for PBC diagnosis	48	41.0%	Medium
Q18—Liver biopsy is mandatory for PBC diagnosis.	116	99.1%	Very good
Q19—The first-line treatment for PBC is ursodeoxycholic acid (UDCA).	79	67.5%	Good
Q20—All PBC patients respond to UDCA.	75	64.1%	Good
Q21—There are second-line treatment options for PBC.	92	78.6%	Good
Q22—Obeticholic acid and fibrates can be used as second-line treatments.	62	53.0%	Medium
Q23—Treatment response should be evaluated 6–12 months after starting UDCA.	65	55.6%	Medium
Q24—Antibiotics can be used in PBC treatment	41	35.0%	Medium
Q25—Steroids can be used in PBC treatment.	8	6.8%	Very low
Q26—There is no treatment option for itching in PBC patients	88	75.2%	Good
Q27—Osteoporosis risk is increased in PBC patients.	71	60.7%	Good
Q28—Bone mineral density testing should be performed in PBC patients.	64	54.7%	Medium
General	73.3 (Mean)	62.6% (Mean)	Medium-Good

* Awareness levels are categorized as Very Low (<10%), Low (10–30%), Moderate (31−60%), Good (61−80%), and Very Good (>80%).

**Table 3:** The correct response rates to the PBC awareness survey between the groups.

Specialization	Participants (n)	Median	Mean ± SD
Gastroenterologist	18	91	89.2 ± 10.3
Gastroenterology Fellow	13	85.7	84.3 ± 6.6
General Surgeon	10	80.3	75.3 ± 16.4
Internist	89	78.5	74.6 ± 12.6
Internal medicine trainee	64	75	71.3 ± 13.6
Other Fellows	15	75	71.1 ± 18.1
General Surgery Trainee	10	75	62.1 ± 28.0
Family Medicine Trainee	19	67.8	65.6 ± 9.9
General Practitioner	25	64.2	62.6 ± 18.1
Family Medicine Specialist	6	60.7	65.4 ± 12.3

**Table 4:** Pairwise Comparisons of Awareness Scores Across Medical Specialties Using the Dunn-Bonferroni Post Hoc Procedure.

Group 1	Group 2	Mean Rank Difference	Standard Error	Adjusted *p*-Value *
Gastroenterologist	General Practitioner	98.4	18.5	<0.001
Gastroenterologist	Internal Medicine Specialist	62.1	14.2	<0.001
Gastroenterologist	Family Medicine Resident	84.1	19.3	0.001
Gastroenterology Fellow	Family Medicine Resident	72.1	21.0	0.007
Gastroenterology Fellow	General Practitioner	86.5	20.3	0.019
Internal Medicine Specialist	General Practitioner	36.2	12.8	0.021
Internal Medicine Specialist	Family Medicine Resident	21.9	13.9	0.035

* Significance level remains at 0.05. *p*-values are adjusted for multiple comparisons using the Dunn–Bonferroni method. Medians represent the percentage of correct responses within each specialty.

**Table 5:** The awareness score between participants.

	Female N = 130	Male N = 140	*p*
Awareness Score (0–100) mean, SD	73.4 ± 13.6	74.8 ± 17.2	0.4
	Under 35 years old N: 193	Over 35 years old N: 77	
	73.0 ± 15.5	76.9 ± 15.5	0.5
	Specialist in any field N: 136	Trainee in any field N: 134	
	77.0 ± 13.5	71.7 ± 15.9	0.003
	Graduation < 10 years N: 192	Graduation > 10 years N: 78	
	73.2 ± 14.4	77.2 ± 15.8	0.04
	Working in a UH N: 175	Not working in a UH N: 95	
	74.0 ± 16.1	74.3 ± 14.6	0.8

SD (standard deviation); UH (university hospital).

## Data Availability

The dataset can be made available from the corresponding author by request.

## Abbreviations

The following abbreviations are used in this manuscript:

PBC	Primary Biliary Cholangitis
AMA	Anti-mitochondrial Antibody
ANA	Anti-nuclear Antibody
IQR	Interquartile Range
UDCA	Ursodeoxycholic Acid
OECD	Organisation for Economic Co-Operation and Development
NAFLD	Non-alcoholic Fatty Liver Disease
HCC	Hepatocellular Carcinoma
Q	Question

## Appendix A

The final version of the survey:

⁢

Dear Colleague,

⁢

We are conducting this survey to increase awareness of primary biliary cholangitis (PBC) and to understand how it is evaluated in daily clinical practice. For this purpose, we kindly ask you to answer questions about the general characteristics of the disease, which will take approximately 9–10 min.

⁢

This survey is intended for general practitioners, internal medicine, general surgery, and family medicine residents and specialists, as well as internal medicine subspecialists and their residents. We kindly ask colleagues from other specialties not to participate.

Please do not seek help while answering the questions. To ensure accurate results, it is important that you answer the questions based on your own knowledge and do not go back to change your previous answers after reading the next question.

This survey does not include any personal data such as names or surnames. Thank you very much for your time.

Respectfully,

Dr. Hasan Eruzun

**Demographic Characteristics**

Age:

20–25,

26–30,

31–35,

36–40,

41–45,

46–50,

51–55,

56–60,

60 and above

⁢

Gender:

Female,

Male

⁢

Workplace:

State Hospital,

University/Training and Research Hospital,

Private Hospital or Private Practice,

Family Health Center

⁢

Specialty/Position:

General Practitioner,

Internal Medicine Resident,

Internal Medicine Specialist,

General Surgery Resident,

General Surgery Specialist,

Family Medicine Resident,

Family Medicine Specialist,

Gastroenterology Fellow,

Gastroenterology Specialist,

Other subspecialty residents and specialists

⁢

How many years since you graduated from medical school?

1–5,

6–10,

11–15,

16–20,

20 and above

⁢

How many years in residency?

1–2,

2–3,

3–5,

More than 5

(Residents only)

⁢

How many years working as a specialist?

1–5,

6–10,

11–15,

16–20,

20 and above

(Specialists only)

⁢

Are you familiar with PBC patients?

⁢

Yes/No/I don’t know

**PBC awareness**

**PBC Epidemiology**

1. PBC was formerly known as primary biliary cirrhosis.

(Yes/No/I don’t know)

⁢

2. PBC is more common in women.

(Yes/No/I don’t know)

⁢

3. PBC does not occur in men.

(Yes/No/I don’t know)

⁢

4. The prevalence of PBC in men is increasing.

(Yes/No/I don’t know)

⁢

5. The prognosis in men is worse than in women.

(Yes/No/I don’t know)

⁢

6. Other autoimmune diseases (e.g., autoimmune thyroiditis) can be seen in PBC patients.

(Yes/No/I don’t know)

⁢

7. PBC is a liver-specific disease.

(Yes/No/I don’t know)

⁢

8. PBC is associated with large bile ducts.

(Yes/No/I don’t know)

⁢

9. PBC is associated with small intrahepatic bile ducts.

(Yes/No/I don’t know)

⁢

10. PBC is an autoinflammatory liver disease.

(Yes/No/I don’t know)

⁢

11. PBC does not lead to cirrhosis.

(Yes/No/I don’t know)

⁢

12. PBC does not cause hepatocellular carcinoma.

(Yes/No/I don’t know)

⁢

13. PBC does not cause liver failure.

(Yes/No/I don’t know)

**Diagnosis**

14. Anti-mitochondrial antibody (AMA) positivity plays a critical role in PBC diagnosis. (Yes/No/I don’t know)

⁢

15. Other than AMA, there is no other antibody specific for PBC diagnosis.

(Yes/No/I don’t know)

⁢

16. Fatigue and itching are the most common symptoms of PBC.

(Yes/No/I don’t know)

⁢

17. AMA positivity is mandatory for PBC diagnosis.

(Yes/No/I don’t know)

⁢

18. Liver biopsy is mandatory for PBC diagnosis.

(Yes/No/I don’t know)

**Treatment**

19. The first-line treatment for PBC is ursodeoxycholic acid (UDCA).

(Yes/No/I don’t know)

⁢

20. All PBC patients respond to UDCA.

(Yes/No/I don’t know)

⁢

21. There are second-line treatment options for PBC.

(Yes/No/I don’t know)

⁢

22. Obeticholic acid and fibrates can be used as second-line treatments.

(Yes/No/I don’t know)

⁢

23. Treatment response should be evaluated 6–12 months after starting UDCA.

(Yes/No/I don’t know)

⁢

24. Antibiotics can be used in PBC treatment.

(Yes/No/I don’t know)

⁢

25. Steroids can be used in PBC treatment.

(Yes/No/I don’t know)

⁢

26. There is no treatment option for itching in PBC patients.

(Yes/No/I don’t know)

⁢

27. Osteoporosis risk is increased in PBC patients.

(Yes/No/I don’t know)

⁢

28. Bone mineral density (BMD) testing should be performed in PBC patients.

(Yes/No/I don’t know)
